# Short- and medium-term follow-up of transcatheter closure of perimembranous ventricular septal defects

**DOI:** 10.1186/s12872-019-1188-y

**Published:** 2019-10-16

**Authors:** Hao Li, Youyang Shi, Songyue Zhang, Yue Ren, Xing Rong, Zhenquan Wang, Tianhe Xia, Tingting Wu, Haitang Xu, Yaoyao Yan, Yi Zhan, Maoping Chu, Rongzhou Wu

**Affiliations:** Children’s Heart Center, The Second Affiliated Hospital and Yuying Children’s Hospital, Institute of Cardiovascular Development and Translational Medicine, Wenzhou Medical University, Wenzhou, 325027 Zhejiang China

**Keywords:** Ventricular septal defect, Transcatheter closure, Complications, Short- and midium-term follow-up

## Abstract

**Background:**

Transcatheter closure of perimembranous ventricular septal defects is one of the greatest challenges in interventional cardiology. Short- and midium-term follow-up data for large samples are limited. This report presents our experience with transcatheter closure of perimembranous ventricular septal defects using an occluder.

**Methods:**

Two hundred fifty-three patients included in the database of the Second Affiliated Hospital and Yuying Children’s Hospital from January 2011– December 2015 with transcatheter closure of perimembranous ventricular septal defects and discharged from follow-up. All patients were invited for clinical and transthoracic echocardiography, electrocardiogram, and thoracic radiography check-up.

**Results:**

Device implantation was successful in 252 of 253 patients (99.6%). The median age was 42 months (range 27–216 months). The median follow-up duration was 36 months (range 6–60 months). The mean defect diameter was 3.5 ± 1.4 mm and the mean size of the ventricular septal defect rim below the aortic valve was 3.7 ± 1.8 mm. The mean diameter of the devices used was 4 mm. Thirty-seven patients developed arrhythmia after the procedure and recovered within 24 months; four patients had hemolysis and four had moderate tricuspid valve regurgitation. No other serious adverse event occurred during the follow-up period.

**Conclusion:**

Transcatheter closure of perimembranous ventricular septal defects using an occluder is safe and effective in most patients.

**Electronic supplementary material:**

The online version of this article (10.1186/s12872-019-1188-y) contains supplementary material, which is available to authorized users.

## Background

The ventricular septal defect (VSD) is one of most common congenital heart defects worldwide. Perimembranous ventricular septal defect, involve a more extensive area than normal membranous septum, also known as aortic subvalvular type. The defect is surrounded by membrane, or the upper edge is fibrous and the lower edge is muscular. With advances in imaging and screening of infants, the detection rate of confirmed cases of VSD has increased notably [1]. Almost 45% of VSDs occurring in isolation close spontaneously [2]. However, for patients with medium and larger (perimembranous or muscular) defects, surgical treatment is often recommended.

Admittedly, traditional surgical repair has yielded great results. Nevertheless, residual VSDs are frequently associated with complications, such as reoperation, infection, sternotomy scarring, and even death [3, 4]. In 1987, Lock [5] was the first to use the Rashkind double-umbrella occluder to close a VSD. Since then, transcatheter VSD closure has been used increasingly in selected patients worldwide. Nevertheless, this technique is associated with complications, such as embolization of the device, hemolysis, aortic insufficiency, and complete heart block. Although various centers in China have reported the use of interventional therapy for VSDs, few data on long-term results are available. We assessed 253 patients through periodic follow-up. In this study, we report the short- and midium-term follow-up results of transcatheter closure of perimembranous VSDs using an occluder in 253 selected patients at our medical center.

## Methods

### Study design

This retrospective, descriptive, non-randomized study utilized data from January 2011–December 2015 from a single medical center (The Second Affiliated Hospital and Yuying Children’s Hospital, Institute of Cardiovascular Development and Translational Medicine, Wenzhou Medical University, Wenzhou, Zhejiang). Two hundred fifty-three patients (126 males, 127 females) were included (Table 1, Additional file 2: Table S2). Informed consent was obtained from all patients or their parents. All patients had clinical and echocardiographic evidence of ventricular septal defect (VSD). The position and distance between each valve and the defect should be carefully assessed preoperatively using echocardiography.

**Table 1:** General Characteristics of Patients

	253 patients Female 127 (50.2%)/ Male 126 (49.8%)
Age (months)	Median = 42 (27–216)
0–36	72 (28%)
36–72	132 (52%)
72–108	24 (10%)
108–144	15 (6%)
144–216	10 (4%)
Weight (kg)	Median = 14.75 (9.5–86)
VSD size (narrowest diameter) (mm)	Mean = 3.5 ± 1.4
VSD rim to the aortic valve	Mean = 3.7 ± 1.8
LVEDd (mm)	Mean = 35.6 ± 5.1
TR-PG (mmHg)	Mean = 31.6 ± 11.6
CTR	Mean = 0.533 ± 0.044
VSD type	Perimembranous
Multiple VSDs	43 (17%)

*VSD* Ventricular septal defect, *LVEDd* left ventricular end-diastolic dimension, *TR-PG* tricuspid valve regurgitation pressure gradient, *CTR* cardiothoracic ratio

### Closure protocol

According to the guidelines for interventional therapy via catheter for congenital heart disease, all patients underwent combined intravenous anesthesia and were monitored via electrocardiogram and blood pressure and oxygen saturation measurement. The peritoneal inguinal puncture site was anesthetized locally with 2% lidocaine, and the femoral vein and femoral artery were punctured. Left and right cardiac catheterization and left ventricular and ascending aortic angiography were performed to determine the size, location, shape, and distance from the aortic valve of the defect. The femoral artery, aorta, left ventricle, VSD, right ventricle, and femoral vein were identified. A long sheath was inserted into the left ventricle along the track from the side of the femoral vein, and then fed into the appropriate sealing device in an attempt to plug the defect. Generally, the sealing device must be 1–2 mm larger than the diameter of the defect. Following closure, the patient must be monitored closely for heart murmur via auscultation, left ventricular angiography should be repeated after 15 min to check for residual shunt, and root imaging should be used to assess the presence of aortic valve regurgitation and prolapse. Following successful closure, thoracic echocardiography (TTE) should be used to monitor the occluder location and sharp, and check for residual shunt and tricuspid valve, and to assess the movement of the aortic valve.

### Device details

The following occluder devices were used (Tables 2, 3, 4 and 5): the Amplatzer Membranous VSD Occluder (AGA Medical Corporation) and Amplatzer-like occluders including Membranous Symmetric VSD Occluder (SHSMA Medical Corporation, China) and Cera™ Membranous VSD (Symmetric) Occluder (ShenZhen Lifetech Scientific Inc., China).

**Table 2:** Embolic materials used in transcatheter embolization of VSD

Device type	Company	Nation	Number
Amplatzer Membranous VSD Occluder	AGA Medical Corporation	American	7
Membranous Symmetric VSD Occluder	SHSMA Medical Corporation	China	167
Cera™ Membranous VSD (Symmetric) Occluder	Lifetech Scientific Inc	China	79

**Table 3:** Device type, sizes and recommended sheath size of CeraTM Membranous VSD (Symmetric) Occluder

Type	D (mm)	D1(mm)	D2(mm)	L (mm)	Minimum Recommended Sheath Size
LT-VSD-Sym-04	4	8	8	3	5F
LT-VSD-Sym-05	5	9	9	3	5F
LT-VSD-Sym-06	6	10	10	3	6F
LT-VSD-Sym-07	7	11	11	3	6F
LT-VSD-Sym-08	8	12	12	3	7F
LT-VSD-Sym-10	10	14	14	3	7F
LT-VSD-Sym-12	12	16	16	3	9F
LT-VSD-Sym-14	14	19	19	3	9F
LT-VSD-Sym-16	16	21	21	3	9F
LT-VSD-Sym-18	18	23	23	3	10F
LT-VSD-Sym-20	20	25	25	3	10F
LT-VSD-Sym-22	22	27	27	3	12F
LT-VSD-Sym-24	24	29	29	3	12F

D, Waist Diameter; D1, Right Disc Diameter; D2, Left Disc Diameter; L, Waist Length

**Table 4:** Device type, sizes and recommended sheath size of Membranous Symmetric VSD Occluder

Type	A (mm)	B (mm)	C (mm)	H (mm)	Minimum Recommended Sheath Size
SQFDQ-II 04	4	8	8	3.5	6F
SQFDQ-II 05	5	9	9	4	6F
SQFDQ-II 06	6	10	10	4	6F
SQFDQ-II 07	7	11	11	4	7F
SQFDQ-II 08	8	12	12	4	7F
SQFDQ-II 09	9	13	13	4.5	8F
SQFDQ-II 10	10	14	14	4.5	8F
SQFDQ-II 12	12	16	15	4.5	9F
SQFDQ-II 14	14	18	17	4.5	9F
SQFDQ-II 16	16	22	20	5	10F
SQFDQ-II 18	18	24	22	5	10F
SQFDQ-II 20	20	26	24	5	12F

A, Waist Diameter; B, Left Disc Diameter; C, Right Disc Diameter; H, Waist Length

**Table 5:** Device type, sizes and recommended sheath size of Amplatzer Membranous VSD Occluder

Type	A (mm)	B (mm)	C (mm)	Minimum Recommended Sheath Size
9-VSDMEMB-004	10	8	4	7F
9-VSDMEMB-005	11	9	5	7F
9-VSDMEMB-006	12	10	6	7F
9-VSDMEMB-007	13	11	7	7F
9-VSDMEMB-008	14	12	8	7F
9-VSDMEMB-009	15	13	9	7F
9-VSDMEMB-010	16	14	10	7F
9-VSDMEMB-011	17	15	11	7F
9-VSDMEMB-012	18	16	12	7F
9-VSDMEMB-013	19	17	13	8F
9-VSDMEMB-014	20	18	14	8F
9-VSDMEMB-015	21	19	15	9F
9-VSDMEMB-016	22	20	16	9F
9-VSDMEMB-017	23	21	17	9F
9-VSDMEMB-018	24	22	18	9F

A, Left Disc Diameter; B, Right Disc Diameter; C, Waist Diameter

### Follow-up

Immediately following the procedure, and in months 1, 3, and 6 and years 1, 2, 3, 4, and 5 of follow-up, the shape and orientation of the device and residual shunt were reevaluated through transthoracic echocardiography, electrocardiogram, and thoracic radiography.

### Statistical analysis

Continuous data were expressed as means ± standard deviations with ranges and were compared using analysis of variance (ANOVA) and the student’s t-test, while categorical data are expressed as number and percentage, and were compared using the X^2^-test or Fisher method. All statistical analyses were performed using commercially available software (SPSS version 11.0 for Windows; SPSS Inc., Chicago, IL, USA). *P* values < 0.05 were considered to be significant.

## Results

### Patient groups

The median age at implantation was 42 months (range 27–216 months) and the median weight was 14.75 kg (range 9.5–86 kg). All patients had continuous systolic murmurs and left-to-right shunts with enlarged left ventricular diastolic dimensions. No patient received diuretics or angiotensin-converting enzyme inhibitors before the procedure for there was not any grade II or higher cardiac dysfunction after preoperative evaluation. Transcatheter femoral venous and arterial approaches were used in all patients. The mean VSD size, measured echocardiographically, was 3.5 ± 1.4 mm. Pulmonary-to-systemic flow ratio (Qp/Qs) was 2.3 ± 0.4 (range 1.5–3.0). One patient with Down syndrome underwent transcatheter closure, and a residual shunt, hemolysis, and arrhythmia was found 24 h postoperative. A surgical operation was subsequently performed. (Tables 1 and 6, Additional file 3: Table S3).

**Table 6:** Procedure data

Variables	Values
Procedure time (hours)	Mean = 1.9 ± 0.6
Qp/Qs	Mean = 2.3 ± 0.4
Systolic PA pressure (mmHg)	Mean = 30.6 ± 7.8
Mild pulmonary hypertension; 30–50	*N* = 120
Moderate pulmonary hypertension; 50–70	*N* = 4
Severe pulmonary hypertension; ≥70	*N* = 1
VSD size (mm)	Mean = 3.5 ± 1.4

*Qp/Qs* pulmonary-to-systemic flow ratio, *VSD* Ventricular septal defect, *PA* Pulmonary artery

### Safety and efficacy

**Fig. 1:**
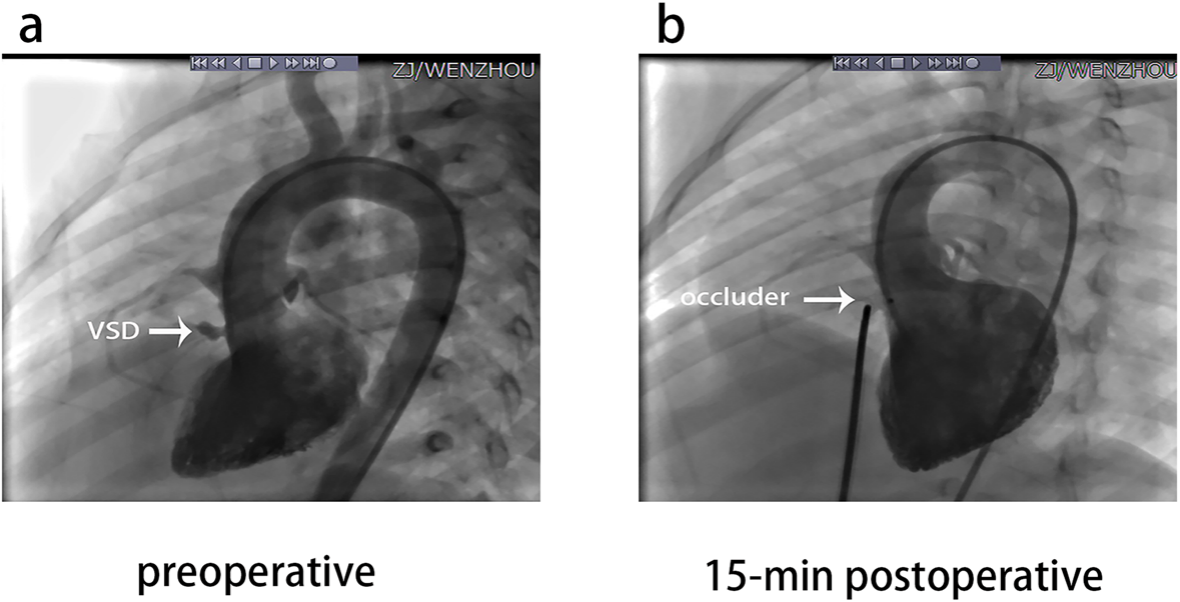
Left ventricular angiography in preoperative and 15-min postoperative. **a** Left ventricular angiography showed the defect with the rim of 3.5 mm (the shunts was designated by arrow); **b** The 5 mm occluder closed the defect without residual shunt

In 252 of 253 (99.6%) procedures, defect closure was successful (Fig. 1). Stable device positioning was demonstrated by angiography and transthoracic echocardiography. Significant shunt reduction was detected in all 252 patients after successful VSD closure, and only minor residual shunts were documented. Procedural data are provided in Table 6. No increase in aortic or tricuspid valve regurgitation was observed postoperatively in any patient.

### Valve regurgitation and residual shunt

**Fig. 2:**
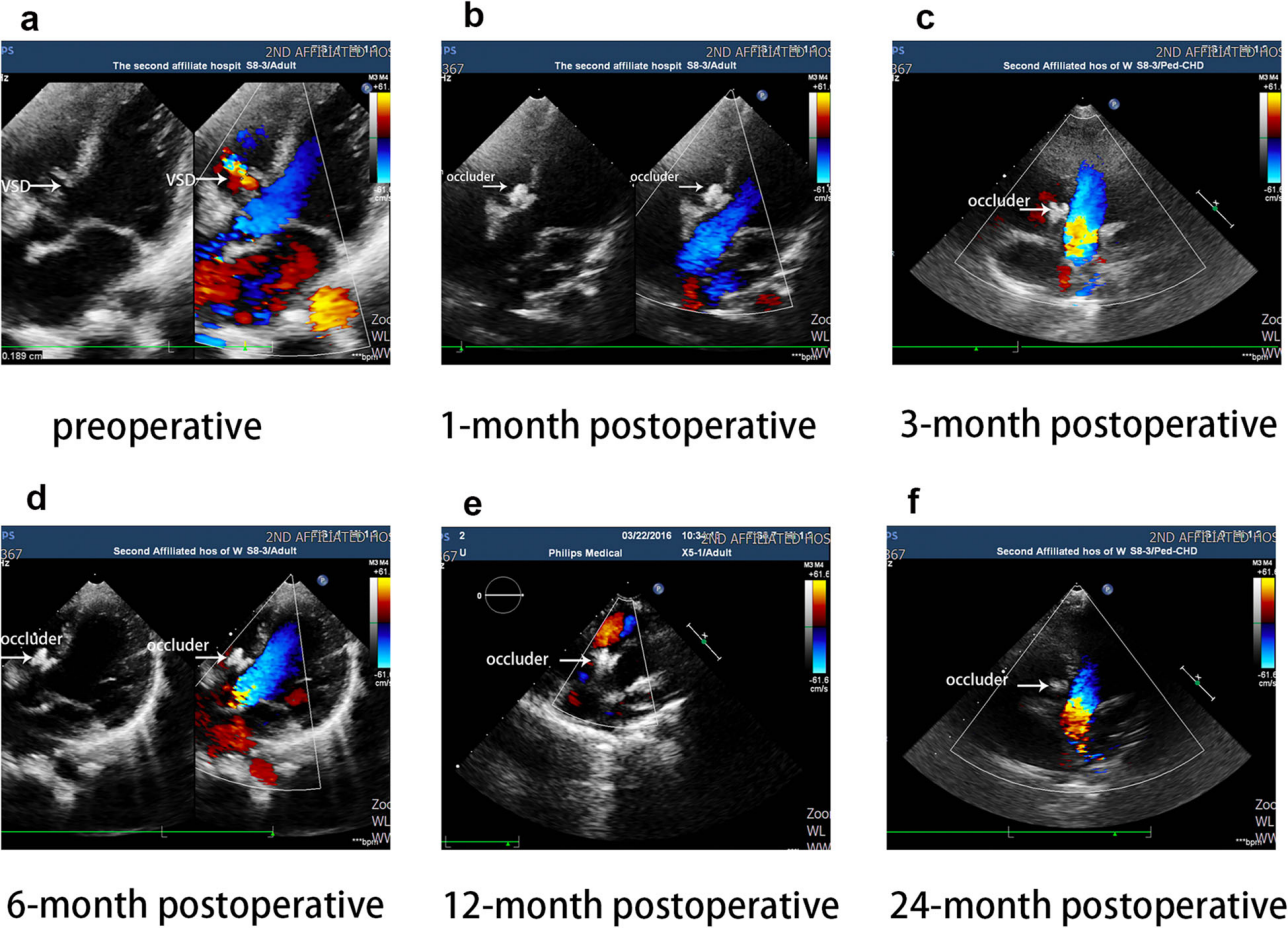
Echocardiography: four-chamher view with VSD, from preoperative to 2 years follow-up (26-month-old patient). VSD:ventricular septal defect; occluder: the device closed the defect without residual shunt. **a**: preoperative(d = 3.5 mm); **b**: at 1 month after the procedure; **c**: at 3 months after the procedure; **d**: at 6 months after the procedure; **e**: at 1 year after the procedure; **f**: at 2 years after the procedure

Pre-operatively, four patients had moderate tricuspid valve regurgitation and nine had mild aortic valve regurgitation. No significant increase or new regurgitation of the aortic or tricuspid valve occurred during or after the procedure. All cases of valve regurgitation had resolved without treatment at the 2-year follow-up evaluation (Fig. 2).

**Fig. 3:**
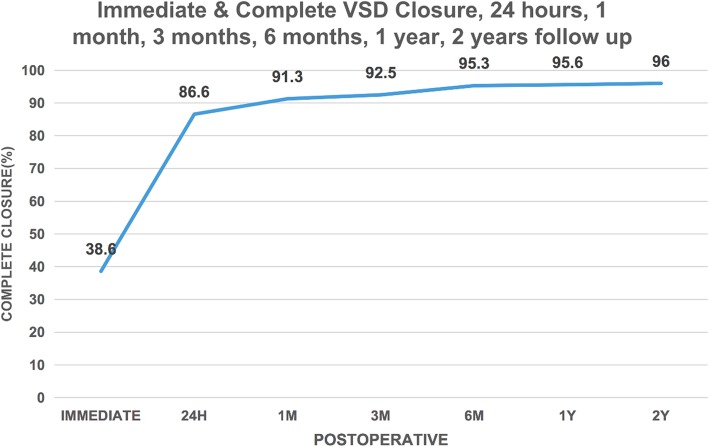
Complete closure percentage immediately on angiography and 24 h, 1 month, 3 months, 6 months, 1 year and 2 years follow up by echocardiography

Closure was achieved with the device in all but one patient. In this case, echocardiography revealed a significant shunt at the center and edge of the occluder, and thoracic surgery was performed. Immediate complete occlusion, determine by angiography, was achieved in 38.6% of patients. Echocardiography performed 24 h postoperatively documented an occlusion rate of 86.6% (219 patients). Echocardiography confirmed the achievement of complete closure in the remaining patients, excluding the patient with a significant shunt described above, at 1 month (*n* = 11), 3 months (*n* = 3), 6 months (*n* = 7), 1 year (*n* = 1), and 2 years (*n* = 1) postoperatively. The occlusion rate at 2 years postoperatively was 96% (Fig. 3).

### Cardiothoracic ratio and tricuspid valve regurgitation pressure gradient

**Fig. 4:**
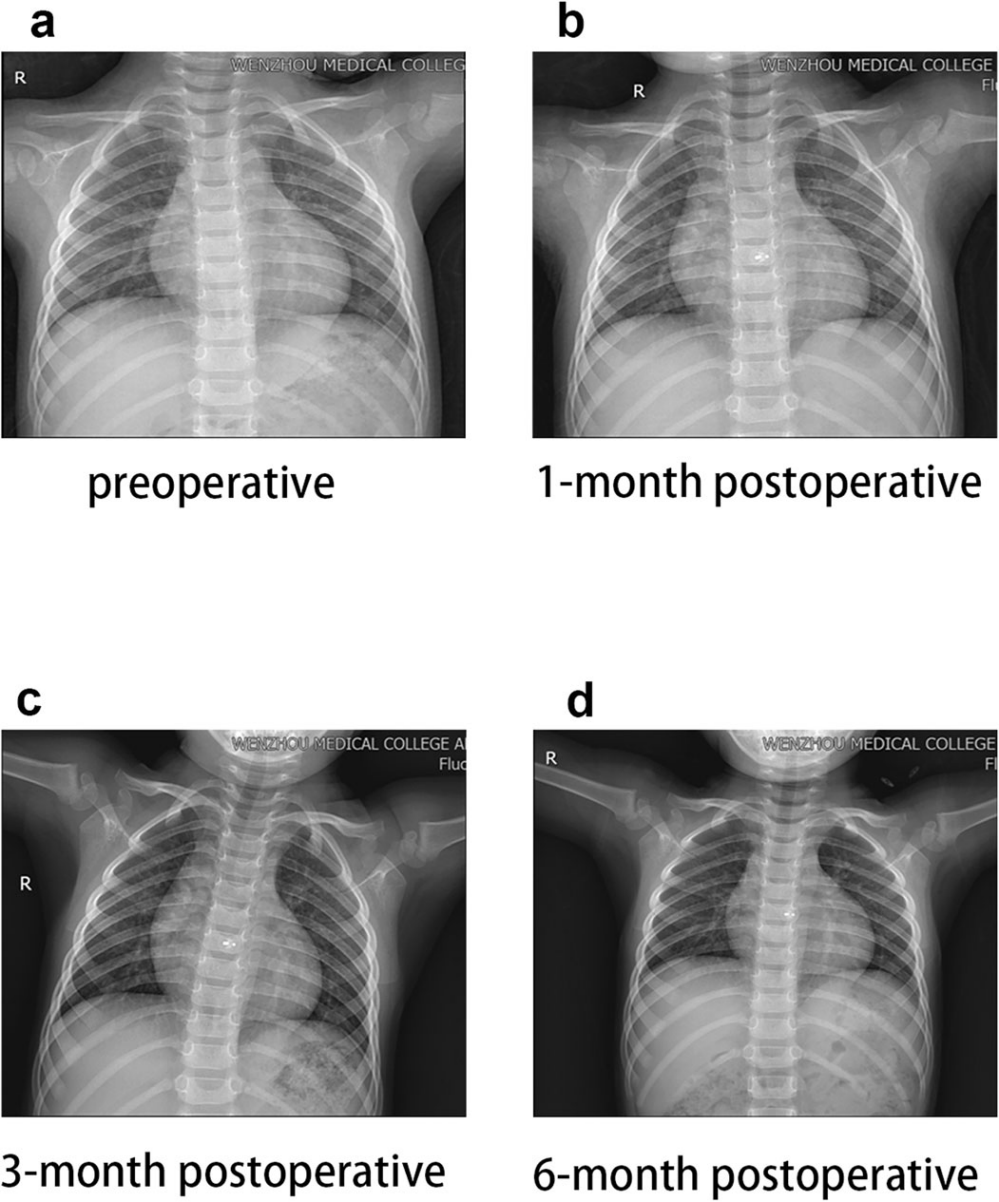
Frontal CTR: from preoperative to 6 months follow-up (26-month-old patient). **a** preoperative; **b** at 1 month after the procedure; **c** at 3 months after the procedure; **d** at 6 months after the procedure

The cardiothoracic ratio decreased gradually from 1 to 24 months postoperatively, and had become stable by the 36-month follow-up evaluation (Fig. 4; Table 7). TTE was used to measure the tricuspid valve regurgitation pressure gradient (TR-PG) for the estimation of pulmonary artery pressure. A significant decrease was detected during follow-up (Table 7).

**Table 7:** Residual shunt, tricuspid valve pressure gradient and cardiothoracic ratio

Time	TR-PG		CTR	
	Mean (mmHg)	*P*-value	Mean	*P*-value
Before closure	31.0 ± 11.6		0.533 ± 0.044	
3-day	25.0 ± 6.8	< 0.05		
1-month	20.9 ± 6.6	< 0.05	0.531 ± 0.052	< 0.05
3-month	21.9 ± 4.0	< 0.05	0.531 ± 0.047	< 0.05
6-month	20.4 ± 5.1	< 0.05	0.527 ± 0.047	< 0.05
12-month	21.7 ± 4.7	< 0.05	0.518 ± 0.042	< 0.05
24-month	22.2 ± 6.6	< 0.05	0.514 ± 0.037	< 0.05
36-month	20.0 ± 2.5	< 0.05	0.505 ± 0.048	NS
48-month	20.3 ± 3.6	< 0.05	0.503 ± 0.028	NS
60-month	21.0 ± 4.7	< 0.05	0.504 ± 0.035	NS

*TR-PG* tricuspid valve regurgitation pressure gradient, *CTR* cardiothoracic ratio

### Complications

No death occurred. Arrhythmia was detected in 37 patients, hemolysis in 4, moderate tricuspid valve regurgitation in 4, and mild aortic valve regurgitation in 9 patients directly after implantation or during follow-up. No significant complication, such as procedure-related obstruction, cardiac tamponade, thrombosis or air embolism, aortic rupture, or increased valve failure, was observed. One significant complication occurred in a 3-year-old boy, who developed hematuria; significant shunt reduction and complete right bundle branch block were detected.

### Hemolysis

No significant hemolysis necessitating device retrieval occurred. Although four (1.6%) patients showed erythrocyte positivity (+++) on routine urine tests during the first 24 h postoperatively, the hemolysis was not sufficiently severe in any case to warrant retrieval of the device. Blood analysis showed high values for hemolysis parameters with no relevant decrease in the hemoglobin concentration. Hemolysis regressed by 5 days postoperatively with hydration and urine alkalization. Blood transfusion was not required in any case.

### Arrhythmia

Minor arrhythmic complications occurred in 37 (17.4%) patients: 14 patients had incomplete right bundle branch block and were treated with a conventional dose of methylprednisolone (20–30 ml/kg/d), which was gradually tapered. 8 had complete right bundle branch block, 3 had left bundle branch block, and 12 had left anterior fascicular block. All patients had recovered, as demonstrated by ECG, at the 24-month follow-up evaluation.

Patients with right bundle branch blocks were treated with a conventional dose of methylprednisolone (1–2 mg/kg, q8h), which was gradually tapered. As left bundle and left anterior branch blocks have been reported to lead to death due to heart failure in China, the methylprednisolone dose can be increased to 20–30 mg/kg/d to relieve ventricular septal edema.

### Follow-up

VSD closure with an occluder was performed successfully in 252 patient. The patients were followed for a median follow-up period of 36 months (range 6–60 months). And the 6 months of follow-up with clinical examination, chest radiography, TTE and ECG was completed in 250 patients (250/253), 1 year in 236 (236/243), 2 years in 190 (190/208), 3 years in 151 (151/169), 4 years in 101 (101/116), 5 years in 32 (32/45) (Table 8). There were 23 patients had lost at follow-up for the following reasons: surgical follow-up for operation, patients from migrant workers’ families going back to their household registration, contact information been replaced. In addition to 9 patients who changed contact information, none of the rest of the patients lost had significant complications such as death or syncope, and they had so far insisted on regular annual follow-up at local children’s heart centers or comprehensive large hospitals. Pre- and postoperatively, TTE was used to detect the tricuspid valve pressure gradient for the estimation of pulmonary artery pressure. TTE showed that the left atrium was reduced compared with preoperative, and gradually increased during follow-up. The TR-PG decreased significantly during the postoperative follow-up period. TTE showed that the LVEDd, RV, and LA had decreased at 3 days postoperatively, decreased further at 1–6 months postoperatively, and had regressed at the 12-month follow-up examination (Table 9).

**Table 8:** Data of follow-up duration

Follow-up duration	Number of followers	Loss of follow-up rate (%)
6 months	250/253	4.3%
1 year	236/243	2.8%
2 years	190/208	8.7%
3 years	151/169	10.7%
4 years	101/116	12.9%
5 years	32/45	28.9%

**Table 9:** Echocardiography Follow-up Datas: preoperative and postoperative

Age (months)	Parameter (mm)	Preoperative	3d	1 m	3 m	6 m	12 m	24 m	36 m	48 m	60 m
< 36	LVEDd	33.4 ± 3.6^*^	31.8 ± 3.2^△^	31.6 ± 3.4^△^	31.6 ± 2.1^△^	31.8 ± 2.4^△^	32.3 ± 2.3	32.4 ± 2.5	35.1 ± 2.0	36.7 ± 1.5	36.9 ± 2.9
	RV	15.0 ± 4.2^*^	15.4 ± 3.9^△^	15.2 ± 3.6^△^	15.4 ± 3.5^△^	16.0 ± 3.6^△^	16.7 ± 4.7	16.5 ± 3.4	16.0 ± 3.4	18.7 ± 3.5	19.2 ± 1.8
	LA	23.0 ± 3.2^*^	22.1 ± 3.1^△^	20.9 ± 3.4^△^	21.1 ± 3.2^△^	21.3 ± 2.8^△^	21.5 ± 3.1	21.3 ± 2.9	23.6 ± 1.9	23.2 ± 4.7	23.6 ± 2.5
	EF	70.2 ± 3.6	70.8 ± 4.5	70.1 ± 3.7	70.9 ± 3.1	69.2 ± 3.7	69.3 ± 4.7	69.2 ± 3.6	71.2 ± 3.2	70.0 ± 3.6	71.2 ± 1.2
36–72	LVEDd	34.7 ± 3.6^*^	32.8 ± 3.3^△^	33.0 ± 3.3^△^	33.0 ± 3.2^△^	33.4 ± 3.1^△^	33.5 ± 3.8	34.9 ± 3.3	35.5 ± 3.8	36.8 ± 2.1	36.5 ± 1.4
	RV	14.4 ± 3.6^*^	14.7 ± 3.6^△^	14.7 ± 3.7^△^	.9 ± 3.3^△^	15.6 ± 3.9^△^	16.1 ± 4.0	16.3 ± 3.6	16.2 ± 4.5	16.4 ± 2.8	16.5 ± 1.7
	LA	22.3 ± 4.3^*^	21.8 ± 3.3^△^	21.3 ± 3.2^△^	21.4 ± 3.1^△^	21.1 ± 2.9^△^	22.2 ± 3.4	22.2 ± 3.8	23.2 ± 3.8	24.7 ± 1.4	23.8 ± 1.3
	EF	70.1 ± 4.3	70.7 ± 4.4	69.3 ± 2.5	69.8 ± 3.5	69.8 ± 3.8	69.8 ± 3.7	69.5 ± 3.6	70.1 ± 2.7	70.8 ± 3.2	69.8 ± 2.4
72-108	LVEDd	39.2 ± 4.3^*^	35.7 ± 4.5^△^	36.8 ± 3.1^△^	38.1 ± 3.9^△^	38.3 ± 3.5^△^	38.0 ± 2.3	38.6 ± 2.1	39.5 ± 1.8	40.1 ± 2.3	41.5 ± 3.6
	RV	15.7 ± 3.9^*^	15.3 ± 3.4^△^	16.3 ± 4.4^△^	18.0 ± 5.7^△^	19.0 ± 5.8^△^	19.1 ± 2.2	18.4 ± 4.	19.3 ± 2.5	19.5 ± 3.1	20.1 ± 3.5
	LA	24.6 ± 3.4^*^	22.7 ± 3.1^△^	23.0 ± 2.6^△^	24.4 ± 3.6^△^	23.8 ± 3.7^△^	23.9 ± 1.9	24.5 ± 3.0	25.1 ± 3.6	26.1 ± 4.5	26.3 ± 5.1
	EF	71.6 ± 4.4	70.0 ± 3.8	69.8 ± 3.4	68.2 ± 3.7	67.1 ± 3.9	68.8 ± 2.2	69.1 ± 3.1	68.9 ± 3.5	69.0 ± 2.0	70.1 ± 2.3
108-144	LVEDd	39.3 ± 2.5^*^	37.6 ± 2.1^△^	37.9 ± 3.7^△^	39.5 ± 3.4^△^	38.2 ± 2.8^△^	38.9 ± 2.0	39.1 ± 2.4	39.5 ± 3.6	40.2 ± 1.8	42.3 ± 3.7
	RV	15.8 ± 2.6^*^	15.8 ± 2.7^△^	16.8 ± 1.9^△^	18.6 ± 5.7^△^	18.3 ± 5.3^△^	18.5 ± 2.7	17.4 ± 3.2	18.9 ± 3.6	18.2 ± 3.4	20.1 ± 3.6
	LA	24.6 ± 3.5^*^	24.7 ± 2.3^△^	24.7 ± 3.6^△^	25.3 ± 3.2^△^	24.1 ± 3.8^△^	24.5 ± 2.8	25.1 ± 3.1	25.7 ± 3.2	26.1 ± 6.7	26.8 ± 3.4
	EF	70.9 ± 3.4	70.3 ± 3.3	67.9 ± 4.2	71.3 ± 3.6	70.2 ± 3.6	70.1 ± 4.5	71.2 ± 3.6	70.9 ± 1.8	68.6 ± 3.6	70.0 ± 6.3
144-216	LVEDd	50.0 ± 2.5^*^	48.5 ± 2.3^△^	47.6 ± 1.5^△^	47.5 ± 2.1^△^	48.1 ± 2.3^△^	48.5 ± 3.4	49.1 ± 3.5	49.5 ± 3.1	50.1 ± 3.5	50.8 ± 3.6
	RV	21.5 ± 5.2^*^	23.7 ± 4.3^△^	22.3 ± 4.4^△^	23.2 ± 3.5^△^	22.2 ± 3.5^△^	22.6 ± 3.6	23.1 ± 3.1	23.5 ± 3.5	24.5 ± 3.1	25.1 ± 4.1
	LA	32.7 ± 2.8^*^	30.5 ± 5.2^△^	32.0 ± 3.4^△^	31.9 ± 3.3^△^	31.7 ± 2.5^△^	32.8 ± 3.1	32.9 ± 3.5	33.1 ± 3.6	33.5 ± 3.1	34.5 ± 2.9
	EF	68.0 ± 1.4	71.1 ± 3.7	66.3 ± 3.1	68.9 ± 3.5	69.9 ± 1.9	69.1 ± 2.3	69.3 ± 2.6	70.1 ± 2.3	68.1 ± 3.1	67.1 ± 2.5

*P*^△^ < 0.05 is statistically different from the previous group, P^*^ < 0.05 is statistically different from normal datas

## Discussion

Ventricular septum defect (VSD) is the most common congenital heart disease, with 40 to 60% of patients with spontaneous closure, mostly in preschool children [6–8].

Surgical closure of VSD has always been a traditional treatment, but there are many complications [6, 9, 10]. With the continuous improvement of occluder instruments in recent years and the selection of appropriate occluder devices according to the shape and size of VSD, the interventional therapy is becoming more and more reliable, safe and effective.

Muscular and perimembranous VSDs have been closed using transcatheter devices at our center since 2002. Use of the transcatheter approach for the treatment of congenital heart diseases is appreciated by patients and their parents because of its decreased psychological impact (avoidance of a large scar), shorter hospital time, reduced pain and discomfort, and avoidance of admission to the intensive care unit [11]. In our study, the results of transcatheter VSD closure with an occluder device were satisfactory: the procedure was successful in 252 of 253 (99.6%) patients, confirming the results reported for interventional VSD closure in other published studies.

The complete VSD closure rate immediately after the procedure was 86.6%, as documented by left ventricular angiography and TTE. This rate increased to 95.3% at the 6-month follow-up evaluation and 96% at the 2-year follow-up evaluation. The follow-up was remained for those children with residual shunts, and most residual shunts resolve postoperatively. These good closure rates are similar to those obtained in other studies of transcatheter VSD closure. Residual shunt is commonly seen in cases of multiple VSDs, as the occluder size must be appropriate for complete defect coverage. Thus the location of the occlusion and the type of occluder should be flexibly selected according to the specific conditions. The general principle to achieve the goal of completely closure of the defect without affecting the function of the aortic valve and the right atrioventricular valve, as well as to reduce the occurrence of complications.

Hemolysis occurred in 4 (1.6%) patients in this study, in whom the mean VSD diameter was 8.1 ± 1.3 mm. All four of these patients had residual shunts. Postoperative hemolysis occurred in association with large defects, high shunt pressure, and residual shunt after closure. Squeezing and collision associated with rapid blood flow through the occluder can cause the mechanical rupture of red blood cells, leading to hemolysis. In these cases, aspirin was stopped and 5% sodium bicarbonate solution (5 ml/kg/d) was used for hydration and alkalization, after which three patients returned to normal after 5 days. The remaining patient developed severe hemolysis accompanied by arrhythmia and residual shunt, necessitating removal of the occluder via surgical thoracotomy.

Thirty-seven (17.4%) patients developed arrhythmia postoperatively. Patients with right bundle branch blocks were treated with a conventional dose of methylprednisolone (1–2 ml/kg, q8h), which was gradually tapered. As left bundle and left anterior branch blocks have been reported to lead to death due to heart failure in China, the methylprednisolone dose can be increased to 20–30 ml/kg/d to relieve ventricular septal edema. Conduction block is related to oppression of the left and right bundle branches due to repeated stimulation of the right ventricular surface at the defect edge by the intraoperative catheter, leading to inflammation of the marginal tissue, edema, and/or exudation. Therefore, excessive long-term intracardiac procedures should be avoided, and the occluder should be slightly larger than the defect diameter of 1–2 mm to avoid compression of the irritated edge tissue (which would lead to tissue edema), thereby preventing arrhythmia. However, evaluating patients with postoperative arrhythmia, ECG monitoring and not 24 h Holter monitoring are limited.

For valve regurgitation, valve insufficiency is associated with the use of an oversized occluder or an insufficient distance from the occluder edge to the valve. A left ventricular disc diameter of the occluder exceeding 50% of the circumference of the subaortic outflow tract has been reported to lead to outflow tract deformation, and thus to valve insufficiency [12]. Therefore, the position and distance between each valve and the defect should be carefully assessed preoperatively using echocardiography. Care should be taken during operation to avoid serious reflux or prolapse, which can damage the valve.

Postoperative follow-up chest radiography showed gradual reduction of the cardiothoracic ratio, with eventual stabilization at 50%, which is close to the normal physiological index. TTE performed immediately after occlusion and 6 months postoperatively showed gradual reduction of the left ventricular end diastolic diameter, right ventricular diameter, and left atrial diameter, indicating smaller inner diameter of the heart cavity and the restored heart structure. At the 12-month follow-up evaluation, the inner diameter indexes of patients’ hearts had increased, but were consistent with the normal developmental ranges in all age groups, suggesting the improvement of hemodynamics. The postoperative TR-PG was significantly lower than preoperatively, suggesting that the pulmonary artery pressure had decreased. The pre- and postoperative left ventricular ejection fraction values did not differ significantly, likely due to the relatively small sizes of defects in this sample.

TTE showed that the left atrium of patients was reduced compared with preoperative, and gradually increased during follow-up. Preoperative left ventricular volume overload and heart enlargement was detected with TTE; postoperatively, due to disappear shunt, the load of the heart was cut down and the heart became smaller than preoperatively. During the follow-up period, the heart became larger due to children growth. For children with growth and development, it is more intuitive to use the z-values to evaluate the heart. However, the z-values commonly used in the world are mainly applicable to Western countries such as Europe and the United States, while these are not suitable for East Asian Chinese. Recently, many centers are now gradually conducting research on the application of Z-values for children’s TTE indicators in East Asian countries such as China (Additional file 1: Table S1).

## Conclusion

Transcatheter closure of VSDs is safe and effective, with a high success rate and few complications. However, indications must be strictly controlled, and the appropriate size of occluder should be selected based on angiographic findings. Patients should be monitored carefully intraoperatively via echocardiography. Further studies are needed to more fully investigate the longer term effects of this procedure.

### Limitation

Holter 24-h monitoring for patients with postoperative arrhythmias is superior to ECG follow-up. Very few patients lost follow-up. Lack of normal echocardiography control.

## Additional files

Additional file 1: **Table S1.** Echocardiography Follow-up Datas: preoperative and postoperative. (DOCX 16 kb)

Additional file 2: **Table S2.** Size of Perimembranous ventricular septal defect. (DOCX 15 kb)

Additional file 3: **Table S3.** Data for pulmonary artery pressure. (DOCX 15 kb)

## Data Availability

The datasets generated during and/or analysed during the current study are not publicly available due to protecting participant confidentiality but are available from the corresponding author on reasonable request.

## Abbreviations

ECG: Electrocardiogram
EF: Ejection fraction
LA: Left atrium
LVEDd: Left ventricular end-diastolic diameter
PA: Pulmonary artery
RV: Right ventricular
TR-PG: Tricuspid valve regurgitation pressure gradient
TTE: Transthoracic echocardiography
VSD: Ventricular septal defects
