# Supplementary material for: Short- and medium-term follow-up of transcatheter closure of perimembranous ventricular septal defects
Source: BMC Cardiovasc Disord. 2019 Oct 16;19:222. doi: 10.1186/s12872-019-1188-y (PMC6794751; doi:10.1186/s12872-019-1188-y)
Supplement: Supplementary file 1 — Table S1. Echocardiography Follow-up Datas: preoperative and postoperative. (DOCX 16 kb) [file 12872_2019_1188_MOESM1_ESM.docx]

**S****upplement 1.** Echocardiography Follow-up Datas: preoperative and postoperative

| Parameter    (mm) | Preoperative | 3d | 1m | 3m | 6m | 12m | 24m | 36m | 48m | 60m |
| --- | --- | --- | --- | --- | --- | --- | --- | --- | --- | --- |
| RV | -0.24±0.81 | -0.29±0.98 | -0.50±1.37 | -0.33±0.84 | -0.38±0.68 | -0.51±0.75 | -0.52±0.69 | -0.36±0.43 | -0.42±0.51 | -0.50±0.71 |
| LA | 1.04±1.07 | 0.98±0.9 | 0.54±1.33 | 1.18±1.14 | 0.44±1.15 | 0.71±0.63 | 1.20±1.11 | 1.23±1.2 | 1.21±1.14 | 1.19±1.15 |
| LVEDd | 0.42±1.37 | -0.4±1.13 | -0.25±1.57 | -0.29±1.09 | -0.40±1.18 | -0.02±0.8 | 0.48±1.10 | 0.52±1.09 | 0.54±1.11 | 0.54±0.90 |
