# Supplementary material for: Short- and medium-term follow-up of transcatheter closure of perimembranous ventricular septal defects
Source: BMC Cardiovasc Disord. 2019 Oct 16;19:222. doi: 10.1186/s12872-019-1188-y (PMC6794751; doi:10.1186/s12872-019-1188-y)
Supplement: Supplementary file 2 — Table S2. Size of Perimembranous ventricular septal defect. (DOCX 15 kb) [file 12872_2019_1188_MOESM2_ESM.docx]

**Supplement 2.** Size of Perimembranous ventricular septal defect

| Age (months) | Number of cases | VSD size (mm) |
| --- | --- | --- |
| [0,36) | 72 | Mean = 4.1±2.2 |
| [36,72) | 132 | Mean = 4.0±1.7 |
| [72,108) | 24 | Mean = 3.2±0.7 |
| [108,144) | 15 | Mean = 4.0±1.2 |
| [144,216) | 10 | Mean = 5.0±1.8 |
