# Supplementary material for: Short- and medium-term follow-up of transcatheter closure of perimembranous ventricular septal defects
Source: BMC Cardiovasc Disord. 2019 Oct 16;19:222. doi: 10.1186/s12872-019-1188-y (PMC6794751; doi:10.1186/s12872-019-1188-y)
Supplement: Supplementary file 3 — Table S3. Data for pulmonary artery pressure. (DOCX 15 kb) [file 12872_2019_1188_MOESM3_ESM.docx]

**Supplement 3.** Data for pulmonary artery pressure

| Variables (catheterization) | Values (mmHg) | Number of cases |
| --- | --- | --- |
| Systolic PA pressure | Mean = 30.6 ± 7.8 | 253 |
| Mild pulmonary hypertension | [30,50) | 120 |
| Moderate pulmonary hypertension | [50,70) | 4 |
| Severe pulmonary hypertension | ≥70 | 1 |
